# Application of Robotic Recovery Techniques to Stroke Survivors—Bibliometric Analysis

**DOI:** 10.3390/jpm12122066

**Published:** 2022-12-14

**Authors:** Diana Uivarosan, Simona Gabriela Bungau, Carmen Delia Nistor-Cseppento, Paul Andrei Negru, Alexa Florina Bungau, Anca Maria Sabau, Delia Mirela Tit, Bogdan Uivaraseanu, Andrei-Flavius Radu

**Affiliations:** 1 Department of Preclinical Disciplines, Faculty of Medicine and Pharmacy, University of Oradea, 410073 Oradea, Romania; 2 Doctoral School of Biological and Biomedical Sciences, University of Oradea, 410087 Oradea, Romania; 3 Department of Pharmacy, Faculty of Medicine and Pharmacy, University of Oradea, 410028 Oradea, Romania; 4 Department of Psycho-Neurosciences and Recovery, Faculty of Medicine and Pharmacy, University of Oradea, 410073 Oradea, Romania; 5 Department of Physical Education, Sport and Physical Therapy, Faculty of Geography, Tourism and Sport, University of Oradea, Oradea 410087, Romania

**Keywords:** robotic application, recovery techniques, stroke survivors, science mapping, bibliometric analysis, VOSviewer

## Abstract

Stroke is a significant disability and death cause worldwide and is conventionally defined as a neurological impairment relating to the intense focal harm of the central nervous system (CNS) by vascular causative components. Although the applicability of robotic rehabilitation is a topic with considerable practical significance because it has produced noticeably higher improvements in motor function than regular (physical and occupational) therapy and exempted the therapists, most of the existing bibliometric papers were not focused on stroke survivors. Additionally, a modular system is designed by joining several medical end-effector devices to a single limb segment, which addresses the issue of potentially dangerous pathological compensatory motions. Searching the Web of Science database, 31,930 papers were identified, and using the VOSviewer software and science mapping technology, data were extracted on the most prolific countries, the connections between them, the most valuable journals according to certain factors, their average year of publication, the most influential papers, and the most relevant topical issues (bubble map of term occurrence). The most prolific country in the analyzed field and over the entire period evaluated (1975–2022) is the United States, and the most prolific journal is Neurorehabilitation and Neural Repair, observing a marked increase in the three periods of scientific interest for this field. The present paper assesses numerous scientific publications to provide, through statistical interpretation of the data, a detailed description of the use of robotic rehabilitation in stroke survivors. The findings may aid scientists, academics, and clinicians in establishing precise goals in the optimization of the management of stroke survivors via robotic rehabilitation, but also through easier access to scientifically validated literature.

## 1. Introduction

Being a leading global contributor to high mortality and morbidity rates, stroke or cerebral vascular accident (CVA) is still a critical neurological disorder that affects thousands of individuals every year [1,2]. Patients who have suffered a stroke and survived commonly encounter symptoms such balance difficulties, impaired vision, paralysis of specific body parts, depression, aphasia, and other disturbances to the cognitive functioning. Stroke survivors continue to have a significant disability that affects how they function independently, understand the environment and connect with others [3,4]. Following the age of 65, stroke is the leading cause of disability, exposing patients to the need of long-term care and recovery requirements. Moreover, it is an etiological component for the development of long-term disability [5]. Due to these medical conditions, stroke is a significant health concern with a serious impact on patients and their caregivers also because of the high number of sequelae [2,5,6].

Worldwide, there are estimated 16 million first-time stroke occurrences each year, which result in 5.7 million deaths [7]. Annually, more than 700,000 Americans have a stroke and 60–75% of these patients will live for more than a year following the event, making up the United States’ population of stroke survivors, numbering about 3 million [8]. According to estimations, this illness will surpass all the other causes of death by the year 2030 [3]. Only around a third of stroke survivors will ever completely recover, even with the best treatment in specialized units [9]. Additionally, stroke is a life-threatening condition with damaging consequences on the motor and intellectual functions that is essential to the public’s health since it entails significant social and financial costs [4,10].

After a stroke, patients are provided a comprehensive, personalized rehabilitation plan that might last an indefinite amount of time, beginning in the intensive care unit, and then continuing at a residence or in a medical recovery clinic. The goal of recovery therapy is to assist stroke survivors to maintain their highest suitable level of physical, cognitive, social, and psychological functioning [11]. Longer and more rigorous training sessions than those achievable with traditional therapy (physical and occupational) are facilitated by rehabilitation robots. Additionally, robot-assisted treadmill training will allow for the monitoring of functional advancements over time as well as the delivery of objective feedback during a single training course [12].

Target-setting, elevated practice, interdisciplinary team management, and task-specific instructions correlated with the mechanisms of stroke are the guiding principles of stroke rehabilitation. The goal of post-stroke recovery is to assist the patient’s reinsertion into society, and the restoration of motor activity is a priority. Emerging neurological rehabilitation technology provides patients with tangible advantages and delivers effective therapies. During their development, each of the following components needs to be carefully assessed [13,14].

Standard rehabilitation has been found to be relatively efficient in enhancing walking ability. However, physiotherapists should frequently exert a lot of physical effort throughout this process [15].

The most promising method for restoring motor control after a stroke is task-specific repeated robot-assisted training of the upper and lower extremities [16]. Technological development has contributed to the production of several robotic devices that contribute to the improvement of the patient’s motor functions and in general to the improvement of the patient’s quality of life, making research in this field expand rapidly [14]. Robotic rehabilitation intervention can provide elevated-dosage and high-intensity exercise, rendering it beneficial for patients with neurological disorders caused by spinal cord disease or stroke. Moreover, robotic systems applied for motor rehabilitation include end-effector and exoskeleton types [12]. Robots used mostly for rehabilitation can be classified as either therapeutic or assistive. Although therapeutic robots offer task-particular instructions, assistive robots are used to provide compensation [17]. The advantages provided by robotic technology include extended training sessions, additional repetitions of exercises, enhanced patient safety, and less physically demanding operation by therapists, which will improve the number of practice cycles by requiring less physical effort [18]. Growing evidence suggests that the use of robots in the stroke population promotes early walking recovery [15,19]. Moreover, an effective therapeutic management involves electromechanical and robotic-assisted gait training, in conjunction with standard rehabilitation, supporting even repetitive practice of gait-like movement in patients who use wheelchairs [20]. Regarding safety, mobility, coordination, and neuromuscular stability, the capacity to resume walking is one of the main issues for stroke survivors. Stroke survivors frequently show considerable spatiotemporal asymmetry and equilibrium impairments that affect their autonomy and life quality. As a result, gait rehabilitation is a crucial focus during post-stroke management. In addition, a variety of robotic walking devices are available that can help improve rehabilitation outcomes [21]. A new type of electromechanically assisted gait training in conjunction with physiotherapy has been demonstrated to enhance the rehabilitation of autonomous walking in stroke survivors, according to a Cochrane meta-analysis of 62 randomized controlled studies with 2440 participants [15].

Table 1 highlights several of the most significant robotic systems evaluated for stroke patients’ rehabilitation.

Several devices have been tested in patients with acute or chronic strokes. According to a recent study on specialists’ perceptions and experiences using robotics to improve the recovery of stroke survivors, the primary benefit of rehabilitation robots was that they enhanced the quantity of therapy and practice following the stroke. The essential qualities are simplicity of use and versatility, while the main challenges were high costs and staffing resources [33].

Although scientific interest in this field is growing due to continuous optimizations in robotic rehabilitation that improve the quality of life of stroke survivors, a bibliometric approach to the topic is lacking.

Bibliometric approaches enable researchers to examine big data sets, can support institution decisions (i.e., funding for research), and can guide the selection of publication outlets. Moreover, examining specific topic trends and patterns within certain journals or collections of journals can be easily achieved with the use of bibliometrics. According to the available research, citations are a reliable measure of influence [34]. Bibliometric analyses on medical topics have continuously evolved and developed significantly in recent years, targeting the management of complex pathologies such as rheumatoid arthritis [35], as well as other possible medical applications of robotics [36,37].

This bibliometric paper aims to present the scientific framework of research in the field of robotic rehabilitation in stroke survivors, a topic less approached in the literature by this type of assessment, providing a systematic overview of the applicability of robotic technology in the medical field, assessing current research trends, and identifying research gaps that can be exploited through new research directions. To achieve this aim, science mapping studies were carried out correlated with general descriptive statistical methods, which through the interpretation of the resulting data provided information on the number of publications in the field and the related countries, the most influential journals, scientific collaboration networks, and the most relevant papers in the field. This facilitates access for researchers, academics, clinicians, and students to scientifically validated data that can be used for setting new research objectives and themes, and for selecting research subfields and papers for future publications, as well as for the optimization of the management of stroke survivors through a highly facile access to the latest developments in the field.

## 2. Materials and Methods

An algorithm has been applied in Web of Science database (W.o.S) [38], as follows: (ALL = (STROKE OR CEREBRAL VASCULAR ACCIDENT)) AND ALL = (Stroke robotic rehabilitation OR Robotic Rehabilitation Therapy OR Rehabilitation robot OR Rehabilitation Technology OR Exoskeleton robot OR Erigo^®^ OR Erigo tilt-table exercise OR Erigo FES OR Robotic Assisted Gait Training OR Robotic Assisted Gait Therapy OR Andago OR Lokomat OR Gait trainer OR Gait training machine OR Armeo^®^ Spring OR Hemiparesis OR Robotic arm OR Brain injury survivors OR Hemiplegia after stroke OR Hemiparesis after stroke OR Motor recovery OR Post-stroke). After application of the search algorithm, 39,844 papers were identified: 39,015 were written in English, 227 in German, 195 in Spanish, and the remainder in other various languages.

**Figure 1:**
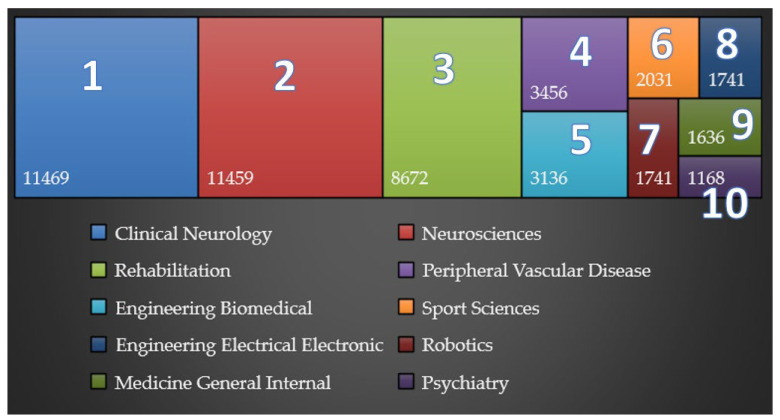
Treemap of the top 10 W.o.S. categories. The numbers from 1 to 10 indicate the domains (whose legend is presented below the figure), in descending order of their relevance (number of articles).

For the present bibliometric analysis, only articles and review articles written in English were selected from the 39,844 identified papers. These search filters reduced the number of papers to 31,930. Figure 1 shows the W.o.S. categories to which most papers were assigned, but 1 paper can be assigned to multiple categories. The most papers were assigned to the following categories: Clinical Neurology, Neurosciences, and Rehabilitation.

The VOSviewer software version 1.6.18 (copyright Nees Jan van Eck, and Ludo Waltman, Leiden, the Netherlands), was used to process the downloaded data using the Export function in the W.o.S. interface. The data required was downloaded as tab delimited files and contained “full record and cited references”. The scientific information is provided by assessments from the first year in which results matching the search algorithm were displayed to the present (1975–2022). For a clearer and more accurate assessment of the evolution over time, the analyses have been divided into 3-time intervals. In the first period assessed (1975–2000), 1641 papers were published, in the second period (2001–2011)—7026, and in the last period (2012–2022)—23,263. During these periods, the most productive countries, and the relationships between them (network map of country co-authorship), the most productive journals and the average year of publication, the most influential papers, and the highest occurrent terms were determined by using science mapping technology.

Highlighting the most productive countries in the field may capture their evolution over the three periods studied, which is correlated with access to technology and funding, opening possibilities for international collaboration to optimize stroke survivors’ management.

Addressing the most prolific journals of the studied topic can assist future authors in selecting journals validated by the large number of articles published, the high number of citations, and the interest in the field analyzed, reducing the risk of rejection or redirection due to inconsistency between the content of the article submitted for publication and the topic covered by the journal.

The total number of citations was used as a dominant measure in assessing the relevance and influence of publications as well as to highlight their acknowledgement in the research community.

Indicating the most relevant keywords facilitates future searches and can form the basis for advanced searches or predefined optimal search algorithms.

**Figure 2:**
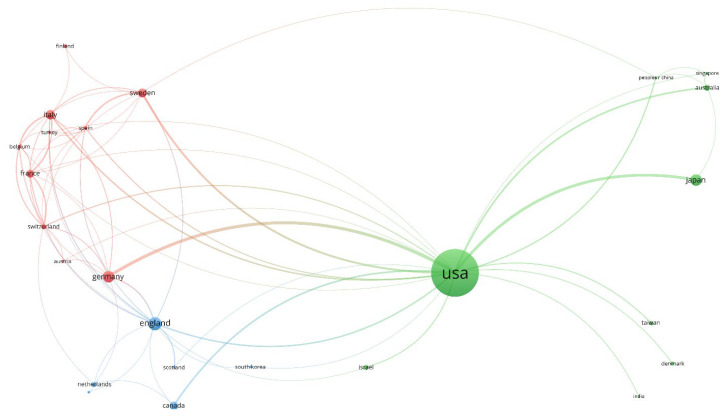
Network map of country co-authorship in the period of 1975–2000.

For the network map of country co-authorship (Figure 2), the size of the bubble is directly proportional to the number of papers published by that country, the thickness of the line connecting two countries is directly proportional to the degree of collaboration between the two countries, and the color of the bubble indicates the cluster to which the country has been assigned. Usually, countries that are in the same cluster show a stronger collaborative relationship.

**Figure 3:**
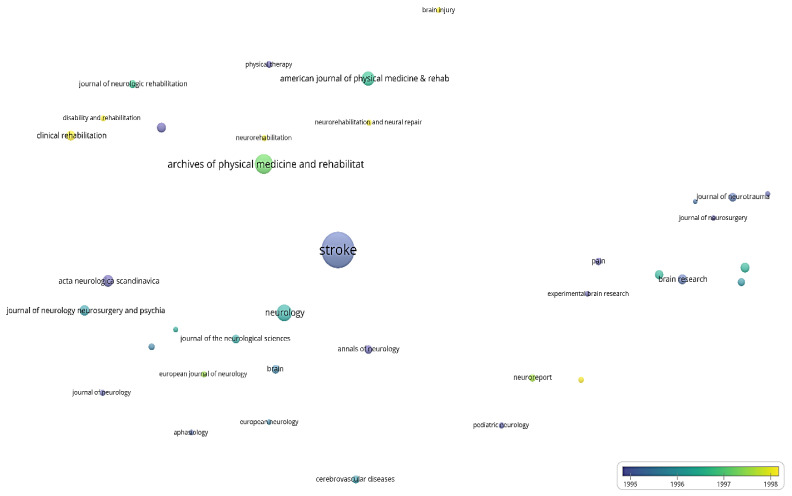
Bubble map of the average publication year in the period of 1975–2000.

For the bubble map showing the average year of publication of journals (Figure 3), the size of the bubble is directly proportional to the number of papers published by the journal and the color of the bubble indicates the average year of publication.

**Figure 4:**
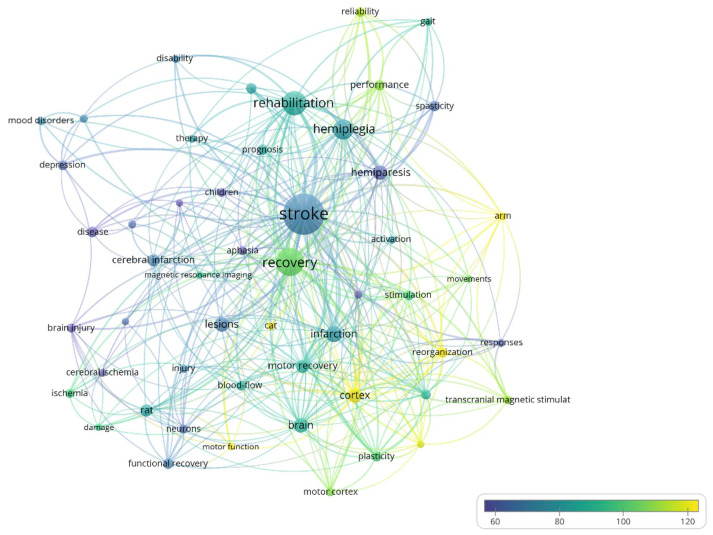
Bubble map of high frequency terms in the field between 1975–2000.

The size of the bubble in the case of the term bubble map (Figure 4) indicates the number of occurrences of the respective term and the color of the bubble represents the average number of citations that the papers including that word receive.

## 3. Results

### 3.1. Period between 1975–2000

#### 3.1.1. Evaluation of the Most Productive Countries in the Field

A number of 49 countries have been identified as contributing to the scientific output during this period. The most prolific country in this period is the United States with 685 published papers and an average citation/paper of 81.48. Table 2 shows the top 10 most prolific countries in terms of the number of published papers. If the ranking was conducted by the top 10 most prolific countries by average citation/paper, Sweden would rank first, indicating that although the number of papers was relatively small (62), their impact was significant.

The total link strength (TLS) feature is a common weighting factor that shows the overall linkage intensity of two objects. In the present paper, the co-authorship relationships between two specific countries are measured overall by the TLS attribute. The countries that collaborated most often are the US with Germany (TLS 15) followed by the US with Japan (13) and the United States with Sweden (8).

The network map of country co-authorship is displayed in Figure 2. The map includes all countries with at least 5 published papers. The 25 countries that met this criterion are grouped in 3 clusters:

The red cluster comprises 10 countries and is led by Germany in terms of the number of papers published;

The green cluster comprises 9 countries and is led by the United States in terms of the number of papers published;

The red cluster comprises 6 countries and is led by England in terms of the number of papers published.

#### 3.1.2. Assessment of the Most Prolific Journals in the Field

A total of 388 bibliographic resources were identified as having published during this period. The most prolific resource during this period was Stroke magazine, with 170 published papers and an average citation/paper of 118.19. The Scandinavian journal of rehabilitation medicine is also in the top 10 most prolific journals due to the high average citation/paper (165.27). Table 3 presents the top 10 prolific journals in the evaluated field.

The average publication year of journals with at least 10 papers published is shown in Figure 3. The number of bibliographic resources meeting this criterion is 37. The most prolific journal of this period, Stroke, has an average publication year of 1995.22; the second most prolific source in terms of papers published, Neurology, has an average publication year of 1996.21. The following journals published the most papers towards the end of the period: Clinical rehabilitation (1998.93), Disability and rehabilitation (1998.20), Neurorehabilitation (1998.21), Neurorehabilitation and neural repair (1999.79), and Journal of cerebral blood flow and metabolism (1998.29).

#### 3.1.3. Citation Analysis of Publications between 1975–2000

A total of 1641 publications were identified during this period, of which 1568 were classified as original research articles and 73 as review articles. Table 4 shows the top 10 most cited papers. The most cited article of this period was published by Fugl-Meyer et al. in 1975 in the Scandinavian journal of rehabilitation medicine, the 10th most productive journal of this period. The second most cited article is a sensitive and reliable locomotor rating-scale for open-field testing in rats, published by Basso et al. in 1995 in the Journal of Neurotrauma.

#### 3.1.4. Term Map and Network Map of Term Co-Occurrence

Figure 4 shows the bubble map of words that have high occurrences during the evaluated period. The minimum number of occurrences a word must have to be represented is 30. The map contains 55 words, of which the following have a high number of occurrences: stroke (491 occurrences, average citations/paper 71.06), recovery (270, 102.79), rehabilitation (211, 82.27), hemiplegia (158, 78.51), and infarction (107, 78.50). The words that have a high average citations/paper are the following: cortex (91, 120.14), reorganization (47, 125.21), and functional reorganization (33, 115.97).

The size of the node is directly proportional to the number of occurrences of the word, the thickness of the line connecting two terms is directly proportional to the strength of the link between the two words (how often they appear together in a paper), and the color of the node indicates the average number of citations across all papers that include that keyword.

### 3.2. Period between 2001–2011

#### 3.2.1. Evaluation of the Most Productive Countries in the Field

The number of countries that published papers during the evaluated period increased to 86. The United States remains in first place in terms of the number of papers published (2902). Table 5 shows the most productive countries of this period. In terms of the average number of citations per paper, Germany ranks first (93.35).

**Figure 5:**
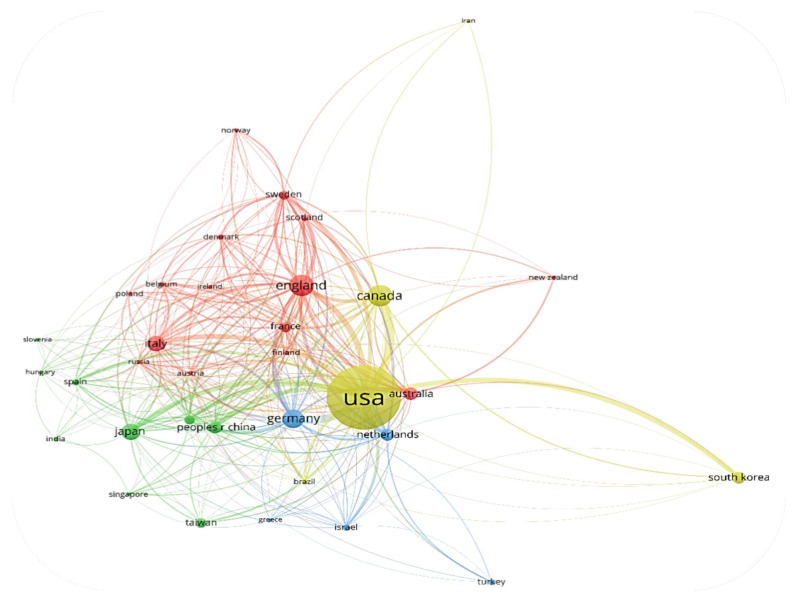
Network map of country co-authorship in the period between 2001–2011.

Figure 5 shows the network map of country co-authorship. The map includes all countries with at least 20 published papers, 34 countries being included, and divided into 4 clusters, as follows:

- The red cluster, which includes 15 countries and is led by England in terms of number of published papers;
- The green cluster comprises 9 countries and is led by Japan in terms of number of published papers;
- The blue cluster comprises 5 countries and is led by Germany in terms of number of published papers;
- The yellow cluster comprises 5 countries and is led by the United States in terms of number of published papers.

The degree of collaboration of the most prolific countries is shown in Table 4 and is represented by the TLS value. The countries that collaborated most often are the United States with Germany (LS-99), followed by the United States with Canada (98) and the United States with England (60).

#### 3.2.2. Assessment of the Most Prolific Journals in the Field

The number of journals that published papers during the evaluated period increased to 1085. The most prolific journal of this period is Archives of physical medicine and rehabilitation and has 275 published papers matching the search parameters used. The top 10 most productive journals are shown in Table 6. In terms of the average number of citations/paper in the top 10, the journal Stroke ranks 1st with 257 published papers and an average of 124.94 citations/paper, and the Journal of Neuroscience with 98 published papers and an average of 156.12.

**Figure 6:**
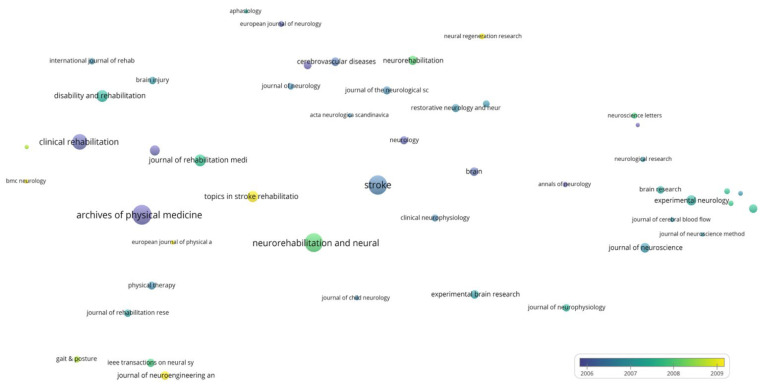
Bubble map of the average publication year between 2001–2011.

The average publication year of journals with at least 30 papers published is shown in Figure 6. Furthermore, 48 journals meet this criterion. The most prolific journal of this period, Archives of physical medicine and rehabilitation, has a mean publication year of 2005.92, indicating that this journal published the majority of papers towards the end of this period. The second most prolific journal in terms of papers published, Stroke, has an average publication year of 2006.45, indicating a more even distribution of papers published/year compared to Archives of physical medicine and rehabilitation. The following journals published the most papers towards the end of the period: Journal of NeuroEngineering and rehabilitation (2009.20), Topics in stroke rehabilitation (2009.07), and Neural regeneration research (2010.24).

#### 3.2.3. Citation Analysis of Publications in the Period between 2001–2011

The total number of papers published during this period is 7026, of which 6410 of them were original research articles and 616 were review articles. The most cited paper of this period was published in 2002 in the journal Clinical neurophysiology by Wolpaw et al. and is entitled “Brain-computer interfaces for communication and control”. The second most cited paper was published in Lancet neurology by Feigin et al. in 2009. The top 10 most cited papers are shown in Table 7.

#### 3.2.4. Term Map and Network Map of Term Co-Occurrence

**Figure 7:**
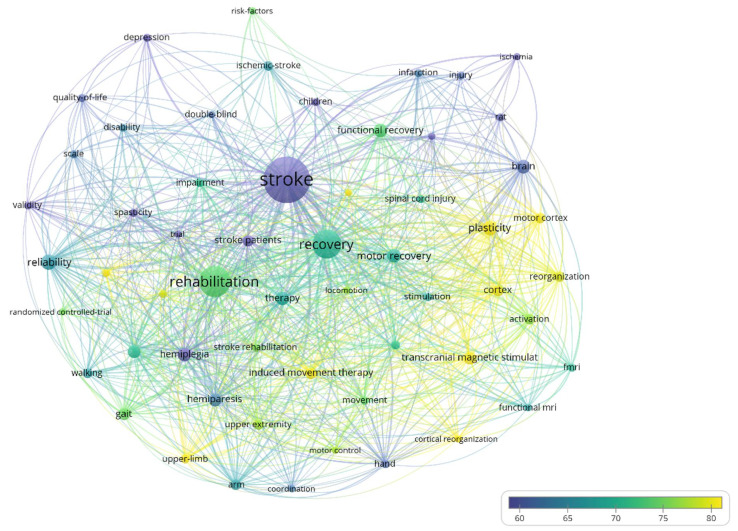
Bubble map of high frequency terms in the field between 2001–2011.

Figure 7 shows the bubble map of words that have high occurrence during the evaluated period. The minimum number of occurrences a word must have to be represented is 150. The map contains 57 words, of which the following have a high number of occurrences: stroke (3117 occurrences, average citations/paper 54.91), rehabilitation (1666, 73.50), recovery (1460, 69.77), reliability (497, 66.20), and plasticity (470, 88.73). The words that have high average citations/paper are the following: motor cortex (292, 81.48), reorganization (270, 78.73), cortex (312, 79.75), transcranial magnetic stimulation (399, 92.85), induced movement therapy (320, 92.28), cortical reorganization (161, 81.08), upper-limb (220, 84.21), and cerebrovascular accident (195, 84.08).

### 3.3. Period between 2012–2022

#### 3.3.1. Evaluation of the Most Productive Countries in the Field

A total of 146 countries contributed to the scientific output of this period. Table 8 shows the most productive countries of this period. In terms of the number of publications, the United States is still in first place, and in terms of the average number of citations/paper in the top 10, Germany ranks first.

**Figure 8:**
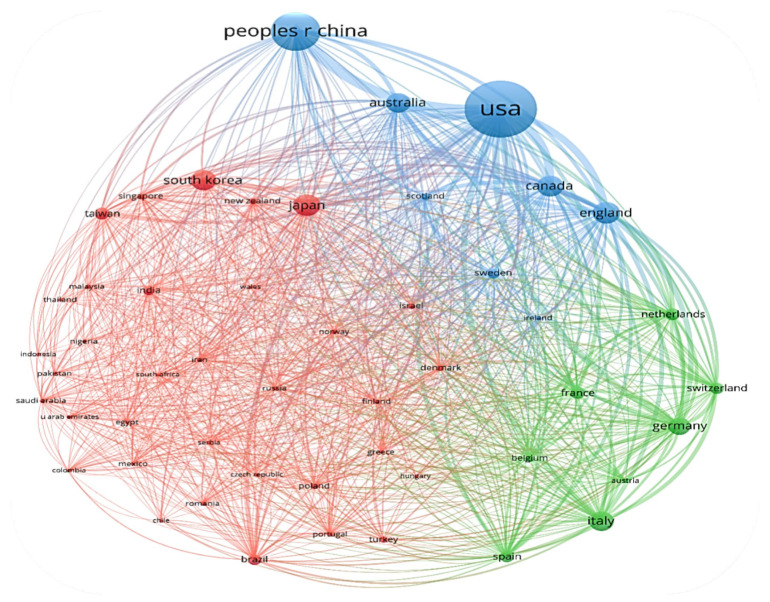
Network map of country co-authorship in the period between 2012–2022.

Figure 8 shows the network map of country co-authorship. The map includes all countries with at least 50 published papers. In total, 50 countries are included, and these are divided into 3 clusters:

- The red cluster, which includes 34 countries and is led by Japan in terms of number of published papers;
- The green cluster comprises 8 countries and is led by Italy in terms of number of published papers;
- The blue cluster comprises 5 countries and is led by the United States in terms of number of published papers.

The degree of collaboration of the most prolific countries is shown in Table 7 and is represented by the TLS value. The countries that collaborated most often are the United States with China (LS − 584) followed by the United States with Canada (370), and the United States with England (325).

#### 3.3.2. Assessment of the Most Prolific Journals in the Field

The 23,263 papers were published in 2590 journals. The journal Frontiers in neurology published the most papers during this period (566) and has an average citation count of 8.19. Table 9 shows the most productive journals of this period.

**Figure 9:**
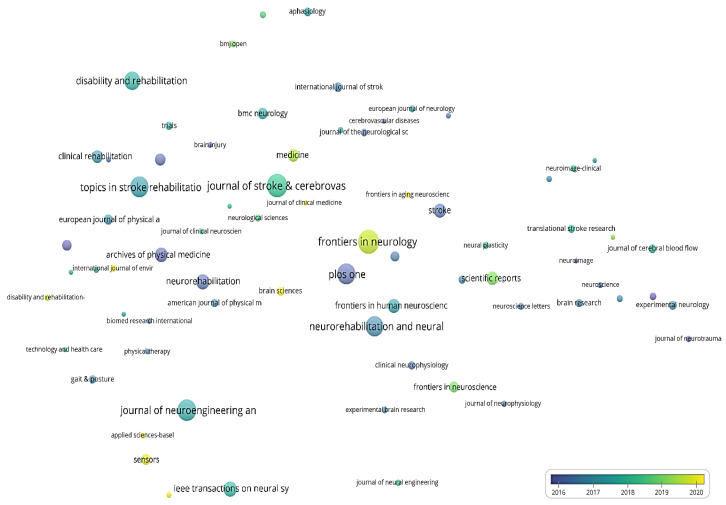
Bubble map of the average publication year between 2012–2022.

The average publication year of journals with at least 60 papers published is shown in Figure 9. The number of journals fulfilling this criterion is 72. The most prolific journal of this period, Frontiers in neurology, has an average publication year of 2019.55, and the second most productive Journal of stroke & cerebrovascular diseases has an average publication year of 2018.18. The following journals published the most papers towards the end of this period: Sensors (2019.75), Applied Sciences-Basel (2022.34), International journal of environmental research and public health (2020.32), Brain sciences (2020.69), and Frontiers in aging neuroscience (2019.85).

#### 3.3.3. Citation Analysis of Publications in the Period 2012–2022

The number of articles published during the evaluated period is 23260, of which 20,309 are classified as original research articles and 2951 as review articles. The most cited article was published by Jovin et al. in 2015 in the New England Journal of Medicine and is entitled “Thrombectomy within 8 Hours after Symptom Onset in Ischemic Stroke”. The top 10 most cited papers are shown in Table 10.

#### 3.3.4. Term Map and Network Map of Term Co-Occurrence

**Figure 10:**
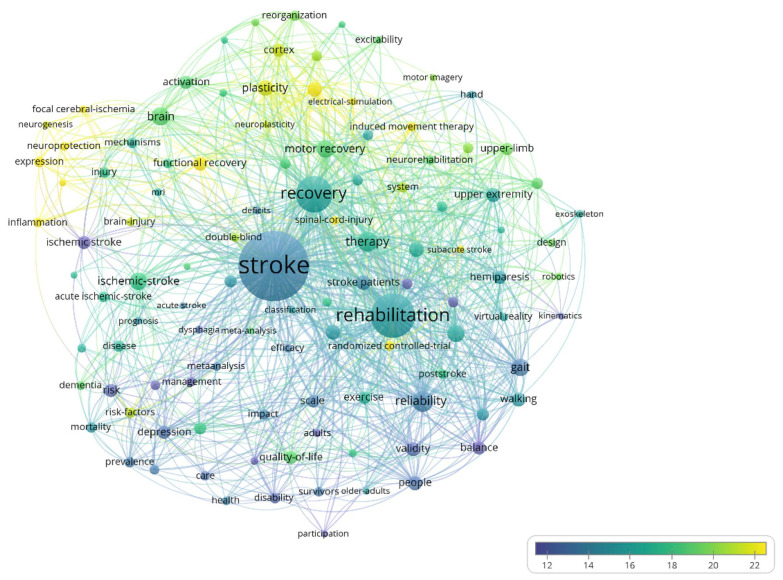
Bubble map of high frequency terms in the field between 2012–2022.

Figure 10 shows the bubble map of words with high occupancy during this period. The minimum number of occurrences a word must have to be represented is 300. In this map, 110 words are represented, of which the following have high occurrence: stroke (12356, 13.99), rehabilitation (5890, 15.44), recovery (4319, 15.95), and therapy (1614, 16.93). The words that have a high average number of citations/papers contained in them are the following: inflammation (474, 21.90), cerebral ischemia (327, 22.10), expression (539, 22.78), neuroprotection (421, 22.14), neurogenesis (323, 21.36), focal cerebral ischemia (389, 30. 78), functional recovery (942, 23.06), transcranial magnetic stimulation (1032, 23.35), electrical stimulation (378, 22.58), induced movement therapy (534, 23.40), spinal cord injury (427, 26.09), and randomized controlled trial (532, 27.81).

## 4. Discussion

The results of the bibliometric analysis confirm the current expectations, observing a growing trend over the years in terms of interest in the applicability of robotics in the field of post-stroke medical rehabilitation, developed in the form of increasing publications in the field.

This continuous development is in close correlation with the technological progress actively involved in the field of medical robotics, presenting certain advantages for the management of various pathologies.

**Figure 11:**
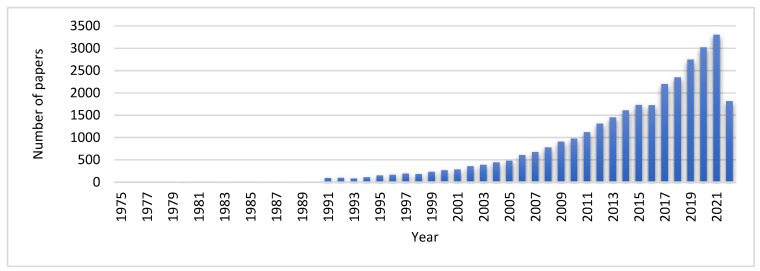
Publication trend of robotic rehabilitation on stroke survivor-related papers correlated with number of citations in 1975–2022.

A total of 31,935 articles were published during the three periods. The number of papers increased gradually from 1975 to 2021. Moreover, the evolution over the years can be observed in Figure 11.

A significant increase in the number of publications is noted, as evidenced by the results of the most prolific country in all three periods analyzed (i.e., United States, with 685 papers in 1975–2000, 2902 papers in 2001–2011, and 6656 papers in 2022). It is worth mentioning that both China and Taiwan were not included in the top 10 most prolific countries in the first two periods evaluated, but in the last period they have shown significant growth, translated by an accelerated applied technological development of these two countries.

In terms of collaborations with other countries, England’s networks are significant, which, although it has a much smaller number of published papers, has a TLS comparable to the United States (30 vs. 94 in the first period, 885 vs. 451 in the second period, and 5183 vs. 3507 in the last period).

The publication pattern has changed over time, with the journals Stroke and Archives of Physical medicine and rehabilitation dominating the first two periods in terms of number of papers published and cited, while in the most recent period, more was published than in the first two combined, and the ranking has changed, with Frontiers in neurology now representing the most prolific journal, closely followed by the Journal of stroke & cerebrovascular diseases, which have developed a lot in recent years and established a complex peer-review system.

Moreover, the IF of journals comprising publications in the field has also increased, with the article with the most citations (*n* = 2925) being published in the New England Journal of Medicine (IF = 176.79) in the last period analyzed. Thus, this topic is becoming increasingly important, and promising results can be transformed into articles in the most valuable journals worldwide. The number of papers published by a journal on a given topic indicates its interest in that scientific subject. Since the number of papers is not an absolute indicator of the quality of the journal, data such as IF and IF without self-citations were also presented.

Table 11 compares the average publication year of the most prolific journals in the three periods analyzed, detailing the information presented above in the corresponding network maps.

The VOSviewer application divides a year into a single unit instead of the customary 12-month division, hence the two decimal places (year.XY), for displaying the average publishing year. The fundamental benefit of a decimal time system is that because the foundation for dividing time and the basis for representing it are essentially the same, hours, minutes, and seconds may all be represented as a single value. As a result, it is easier to understand a timestamp and perform conversions.

The most cited article in the field in the first period analyzed (1975–2000) belongs to Fugl-Meyer et al. It was published in 1975 in the Scandinavian journal of rehabilitation medicine, presenting in-depth approaches for testing hemiplegic patients’ movement patterns, stability, some sensory characteristics, and joint function, thus opening research directions in the field of rehabilitation of stroke survivors and suggesting their importance in improving patients’ quality of life [39].

Nudo et al. is a highly relevant publication in the field, published in 1996 in Science, a journal with an impact factor of 63.714. It represents a paper that has made major contributions to the field through its results. The paper investigated how effective hand rehabilitation following comparable infarcts prevented loss of hand territory near the infarction. Clinical rehabilitation of competent motor function was accompanied by structural remodeling in the intact motor cortex. Furthermore, it was suggested that the unaffected motor cortex may be crucial for motor recovery [42].

Wolpaw et al. published the article with the most citations from the second period (2001–2011), according to the original search algorithm. As technology evolved, brain–computer interfaces for communication and control were evaluated for applicability. It was suggested that the progress of stroke survivors is contingent on the creation of training programs to help users gain and maintain control and in the establishment of optimal algorithms for converting electronic signals into device commands [49].

According to the data obtained from the second period analyzed, robotic rehabilitation techniques for stroke survivors have been successfully optimized and applied. Langhorne et al. (2011) highlighted categories of interventions for stroke survivors identified through systematic reviews or randomized trials in an article published in the Lancet, a journal with an impact factor of 202.731. Robotic rehabilitation was included in the category of novel therapies, highlighting that robotic tools can be used to rehabilitate stroke impairments in a repetitive, dynamic, and task-specific manner [55].

The third and most recent period studied (2012–2022) demonstrates the continued development and improvement of robotic rehabilitation for stroke survivors, which is now integrated into the overall management of these patients.

Hochberg et al. presented in 2012, in a highly cited Nature article, a neurological interface system that automatically translates brain activity into signal amplification for assistive technologies, restoring autonomy and mobility to stroke patients [61]. Furthermore, Winstein et al. published in 2016, in Stroke, a guideline on the rehabilitation and recovery of stroke survivors, including robot-assisted locomotor training through optimal technology systems, as a very important factor in the management of these patients (evidence level A) [21].

An analysis of the most cited articles of the three periods evaluated demonstrates how the development of technology has allowed the transition from manual rehabilitation procedures with lower efficiency and feasibility to the successful application of robotic rehabilitation, with an accompanying increase in scientific interest in this field.

**Figure 12:**
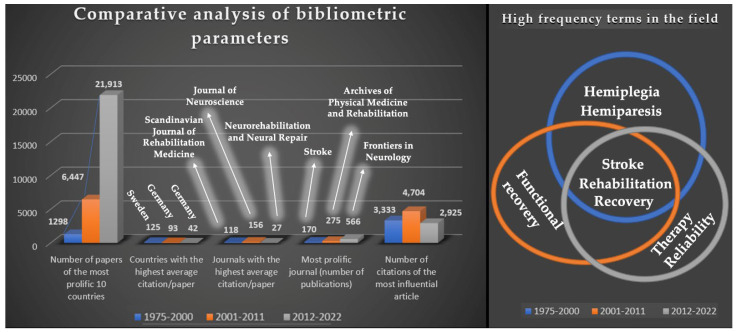
Comparative analysis of bibliometric parameters.

Figure 12 presents a comparative analysis of some relevant bibliometric parameters, which is possible due to the division of the evaluation into three specific periods.

According to the data obtained from the application of the advanced search algorithm, a significant increase in the number of papers published by the 10 most prolific countries corresponding to each analyzed period was observed. The ranking has changed slightly over the three periods, with the most significant impact being an increase in the number of publications in the case of China, which now ranks second in the third period examined in terms of the number of publications.

Countries were ordered in the Results Section 3 based on the number of citations, but for the current comparative analysis, the countries with the highest average citation/paper were highlighted, with Sweden being the most prolific country in the first period studied and Germany in the following two.

It can also be observed that the most prolific journals of the periods analyzed in terms of the number of publications were not the ones with the highest average citations per paper.

In the last analyzed period (in terms of the number of citations in the most impactful articles), a decrease can be identified.

The most constant element remained the high frequency of terms, in all three periods, the most frequently searched terms being “stroke”, “recovery”, and “rehabilitation”, as can be observed in the Venn diagram. One aspect worth mentioning is that the keyword “robotics”, although not a very high frequency term, appeared in the map of the third period evaluated. However, in the last period we can observe an exponential increase in the number of searches for the three terms, proof that the subject is continually growing, access to innovative technologies is easier than in the past, and the funds used by most countries for medical research are increasing year by year.

Bibliometric papers have several shortcomings, first considering the large number of articles that cannot be checked manually; therefore, sometimes false positive papers can infiltrate the analyzed data. Secondly, another major disadvantage specific to this type of paper is the fact that only papers written in English were analyzed, so possible valuable papers may be omitted due to the language barrier. Another limitation encountered in this type of paper is the method of classifying the results (i.e., quantitative classification, by number of papers published, citations, etc.). The number of citations of a paper is not a direct indicator of the quality of a paper, and the number of papers published by a journal/country does not provide information about the quality of published papers.

Citation analyses might be biased against excellent research that is presented in highly qualified journals that only a limited percentage of academics examine. Ethics and morality are significant elements that are not always reflected in publishing rates or citation counts. Authors may be using highly frequent self-citations to boost citation counts and could also improperly cite coworkers, supervisors, or journal editors.

Although bibliometric papers have a few limitations, they can serve as a practical guide for scientist, academics, and students.

This information can be a guide in the pre-research phase to choose a research topic or in the pre-publication phase to see which journals are the most prolific and have the best visibility. It also facilitates the creation of international collaborations by presenting existing networks, most of which are optimized, and access to the most prolific papers in the field, from which research directions can be established or protocols can be set up to help solve existing scientific limitations. The main benefit of bibliometric analyses is their impartiality, because the outcomes cannot be significantly changed by the authors and can be precisely reproduced using the same methodology. Moreover, the results provided by the present bibliometric analysis can constitute as tools for clinicians to be more easily informed about the latest developments in the field and to help them in the successful management of post-stroke symptoms in order to improve the field of robotic rehabilitation, as easy access to validated bibliographic resources optimizes the overall management, thus being able to detect the most prolific articles in the field and the limitations that can be transformed into new research directions, opportunities for international collaboration, etc.

This method’s ability to assess a significant number of publications provides an overview of the issue under investigation.

## 5. Conclusions

The present bibliometric paper analyses a large number of papers (31,930) to provide an overview of the relevant scientific literature on the topic “Applicability of robotic rehabilitation therapy to stroke survivors”. For a better understanding of the data presented, they have been divided into three periods. In the first period (1975–2000), a small number of papers (5.14%) matching the search terms were published. In the 2nd period (2001–2011), the number of papers increased considerably (22.00%). The remaining 72.86% were published from 2012 to 2022, demonstrating a considerable increase in publications, correlated with the technological development in the medical field. Using the maps created with VOSviewer as well as the information extracted with this software, future authors can identify the most influential countries, articles, and journals in the field, and by using the bubble map of the term occurrence, they can determine the most relevant topics of interest.

Facilitating access to new and relevant information in the field is a support for clinicians to learn about new approaches from the perspective of robotic rehabilitation and to access solutions to possible shortcomings encountered in practice, all enhancing the management of stroke survivors.

Additionally, by easing the process of selecting and assessing scientific literature sources in this domain, by identifying knowledge gaps, and by proposing future research paths, this research delivers extensive bibliometric data and contributes to enhancing the future potential results.

## Figures and Tables

**Table 1:** Characteristics of robotic systems used in the rehabilitation of stroke survivors.

Robotic Systems/ Commercialization/ Invention Year	Basic Principle	Applications	Evaluation	Ref.
**Lower-limb robotics—exoskeleton type**				
Lokomat/ (Hocoma AG, Switzerland)/ 2001	A robot-driven exoskeleton orthosis comprises of a software-controlled robotic exoskeleton that operates the patient’s legs in an adaptable manner in connection with a body-weight support structure	Individuals with: spinal cord injuries; traumatic brain injuries; non-traumatic brain injuries (including stroke); cerebral palsy (Children and adults); Parkinson’s disease; multiple sclerosis; Guillain-Barré syndrome; post-surgery (meniscus injury, lumbar discectomy, and arthroscopic total knee)	Walking autonomy, speed, endurance, balance; controlling muscular tone and decreasing stiffness cardiovascular implications; physical characteristics; life quality	[15,22]
Erigo Pro/ Hocoma AG, Switzerland/2014	A robot-driven exoskeleton combining verticality and gradual mobilization with functional electrical stimulation	Massive brain injury patients spinal cord injured patients experiencing orthostatic stress; reduce the time spent in intensive care, time spent in hospital and the overall cost of therapy; reduce medical complications associated with immobility and relieves the strain on the therapist) step-like actions improve brain function in a similar way to overground activity	Cardiovascular normalization; quick and safe movement even during acute care	[23,24]
Gait Trainer I/Reha-Stim Medtec GmbH & Co. Kastanienallee 32 Berlin, Germany/2000	End-Effector system-is built on a dual crank and rocker gear system that possesses two foot plates that are placed on two bars, each of which includes rockers and cranks that serve as the propulsion; although the sufferer is using the device, the foot plates accurately replicate the stance and swing stages of walking.	Adjusts the mass center in both the horizontal and vertical directions, replicates the stages of gait, and supports the participants based on their skills.	Rehabilitation of gait in stroke survivors during the acute stage	[25,26]
**Upper-limb robotics**				
Armeo- (Hocoma AG, Switzerland)/2008	Exoskeleton system-allows patients to practice and repeat hand and arm movements to improve their recovery; consists of three unique devices, each of which targets a particular patient need.	Individuals who have experienced strokes, brain trauma, or neurological conditions that affect their hands and arms should have their strength, flexibility, quality of mobility, and rigidity evaluated.	Daily tasks, arm strength, and arm functionality; motor performance action that is accurate; shorter time to complete activities	[27,28,29]
InMotion robot (Massachusetts Institute of Technology, Mit-Manus):	Five effective degrees of freedom are available at the elbow, shoulder, and wrist due to a wrist robotic device with three active degrees of freedom that is attached at the tip of a companion planar robot (MIT-MANUS)	Patients recovering from neurological disorders and accidents benefit from improved upper-extremity motor retraining in patients with all degrees of muscular strength; restores motor control and enhances results	Arm movement, function, and quality of life.	[30,31]
ARMOTION (Reha Technology AG, Switzerland)	It enables data gathering, monitoring, and precise patient performance measurement; it also enables informative and repeatable activities with video feedback in a 2D workspace.	Optimize the therapeutic impact for patients suffering from severe and mild upper-extremity neurological dysfunctions; early-stage patients can safely observe and acquire shoulder and elbow movements with the help of passive therapy methods.	In the management of severe and mild upper extremity neurological dysfunction	[32]

**Table 2:** Top 10 prolific countries in robotic rehabilitation on stroke survivors’ field published in 1975–2000.

Country	Papers	Citations	Average Citation/ Paper	TLS
United States	685	55,811	81.48	94
England	115	11,618	101.03	30
Japan	95	3600	37.89	14
Germany	94	9310	99.04	27
Italy	67	5478	81.76	26
Sweden	62	7769	125.31	27
Canada	59	6600	111.86	8
France	52	4529	87.10	19
Australia	39	1785	45.77	10
Switzerland	30	2117	70.57	18

TLS, total link strength value attributed by VOSviewer.

**Table 3:** The most prolific journals in robotic rehabilitation on stroke survivors’ field throughout 1975–2000.

Journals	No.	C	Average Citation/Paper	IF	IF without Self-Citations	Publisher
Stroke	170	20,092	118.19	10.170	9.344	LWW
Archives of physical medicine and rehabilitation	71	6233	87.79	4.060	3.804	W B SAUNDERS CO-ELSEVIER INC
Neurology	56	5803	103.63	11.800	11.318	LWW
American journal of physical medicine & rehabilitation	46	2361	51.33	3.412	3.176	LWW
Acta neurologica scandinavica	33	1463	44.33	3.915	3.799	WILEY
Journal of neurology neurosurgery and psychiatry	29	1889	65.14	13.661	13.185	BMJ PUBLISHING GROUP
Brain research	28	1371	48.96	3.610	3.556	ELSEVIER
Clinical rehabilitation	28	1366	48.79	2.884	2.796	SAGE PUBLICATIONS LTD
Experimental neurology	26	1525	58.65	5.620	5.347	ACADEMIC PRESS INC ELSEVIER SCIENCE
Scandinavian journal of rehabilitation medicine *	26	4297	165.27	1.333 (2002)	1.333 (2002)	TAYLOR & FRANCIS AS

No., number of papers; C, citations; IF, impact factor; LWW, LIPPINCOTT WILLIAMS & WILKINS; * in 2001, the name was changed to Journal of Rehabilitation Medicine.

**Table 4:** The most influential papers in the field between 1975–2000.

First Author	Title	Journal	IF	C	Ref.
Fugl-Meyer (1975)	Post-stroke hemiplegic patient. 1. Method for evaluation of physical performance	Scandinavian journal of rehabilitation medicine	1.333 (2002)	3333	[39]
Basso (1995)	A sensitive and reliable locomotor rating-scale for open-field testing in rats	Journal of Neurotrauma	4.869	3251	[40]
Shadmehr (1994)	Adaptive representation of dynamics during learning of a motor task	Journal of Neuroscience	6.709	1705	[41]
Nudo (1996)	Neural substrates for the effects of rehabilitative training on motor recovery after ischemic infarct	Science	63.714	1302	[42]
Schallert (2000)	CNS plasticity and assessment of forelimb sensorimotor outcome in unilateral rat models of stroke, cortical ablation, parkinsonism and spinal cord injury	Neuropharmacology	5.273	1030	[43]
Bonita (1988)	Recovery of motor function after stroke	Stroke	10.170	988	[44]
Bracken (1997)	Administration of methylprednisolone for 24 or 48 h or tirilazad mesylate for 48 h in the treatment of acute spinal cord injury—Results of the Third National Acute Spinal Cord Injury Randomized Controlled Trial	JAMA-journal of the American medical association	157.335	964	[45]
Chollet (1991)	The functional-anatomy of motor recovery after stroke in humans—a study with positron emission tomography	Annals of neurology	11.274	872	[46]
Cramer (1997)	A functional MRI study of subjects recovered from hemiparetic stroke	Stroke	10.17	752	[47]
Weiller (1992)	Functional reorganization of the brain in recovery from striatocapsular infarction in man	Annals of neurology	11.274	710	[48]

IF, impact factor; C, citations, Ref, references.

**Table 5:** Top 10 prolific countries in robotic rehabilitation on stroke survivors’ field published in 2001–2011.

Country	Papers	Citations	Average Citation/ Paper	TLS
United States	2902	226,890	78.18	885
Canada	604	43,364	71.79	310
England	601	46,621	77.57	451
Germany	481	44,900	93.35	366
Japan	406	18,725	46.12	164
Italy	389	30,604	78.67	236
Australia	281	17,238	61.35	177
Netherlands	267	24,299	91.01	171
South Korea	265	10,156	38.32	72
China	251	9890	39.40	154

TLS, total link strength value attributed by VOSviewer.

**Table 6:** The most prolific journals in robotic rehabilitation on stroke survivors’ field throughout 2001–2011.

	No.	C	Average Citation/Paper	IF	IF without Self-Citations	Publisher
Archives of physical medicine and rehabilitation	275	22,633	82.30	4.060	3.804	W B SAUNDERS CO-ELSEVIER INC
Stroke	257	32,110	124.94	10.17	9.344	LWW
Neurorehabilitation and neural repair	249	21,311	85.59	4.895	4.602	SAGE PUBLICATIONS INC
Clinical rehabilitation	193	10,054	52.09	2.884	2.796	SAGE PUBLICATIONS INC
Journal of rehabilitation medicine *	132	7560	57.27	3.959	3.777	FOUNDATION REHABILITATION INFORMATION
Disability and rehabilitation	128	5844	45.66	2.439	2.182	TAYLOR & FRANCIS LTD
Topics in stroke rehabilitation	124	4165	33.59	2.177	2.113	TAYLOR & FRANCIS LTD
American journal of physical medicine & rehabilitation	105	4121	39.25	3.412	3.176	LWW
Experimental neurology	103	7584	73.63	5.620	5.347	ACADEMIC PRESS INC ELSEVIER SCIENCE
Journal of neuroscience	98	15,300	156.12	6.709	6.454	SOC NEUROSCIENCE

No., number of papers; C, citations; IF, impact factor; LWW, LIPPINCOTT WILLIAMS & WILKINS; * previously named Scandinavian journal of rehabilitation medicine.

**Table 7:** The most influential papers in the field between 2001–2011.

First Author	Title	Journal	IF	C	Ref.
Wolpaw (2002)	Brain-computer interfaces for communication and control	Clinical neurophysiology	4.861	4707	[49]
Feigin (2009)	Worldwide stroke incidence and early case fatality reported in 56 population-based studies: a systematic review	Lancet neurology	59.935	1727	[50]
Bouhassira (2005)	Comparison of pain syndromes associated with nervous or somatic lesions and development of a new neuropathic pain diagnostic questionnaire (DN4)	Pain	7.926	1393	[51]
Cogan (2008)	Neural stimulation and recording electrodes	Annual review of biomedical engineering	11.324	1321	[52]
Chen (2001)	Therapeutic benefit of intravenous administration of bone marrow stromal cells after cerebral ischemia in rats	Stroke	10.170	1309	[53]
Cabeza (2002)	Aging gracefully: Compensatory brain activity in high-performing older adults	Neuroimage	7.400	1299	[54]
Langhorne (2011)	Stroke Care 2 Stroke rehabilitation	Lancet	202.731	1290	[55]
Kleim (2008)	Principles of experience-dependent neural plasticity: Implications for rehabilitation after brain damage	Journal of speech language and hearing research	2.674	1178	[56]
Langhorne (2009)	Motor recovery after stroke: a systematic review	Lancet neurology	59.935	1138	[57]
Murphy (2009)	Plasticity during stroke recovery: from synapse to behaviour	Nature reviews neuroscience	38.755	1100	[58]

IF, impact factor; C, citations.

**Table 8:** Most prolific countries in robotic rehabilitation on stroke survivors’ field published in 2012–2022.

Country	Papers	Citations	Average Citation/ Paper	TLS
United States	6656	165,666	24.89	5183
China	3899	56,938	14.60	2371
England	1682	52,805	31.39	3507
Japan	1670	33,000	19.76	1549
Canada	1625	50,447	31.04	2511
South Korea	1494	29,322	19.63	1257
Italy	1440	44,803	31.11	2541
Australia	1436	40,921	28.50	2563
Germany	1268	53,450	42.15	2861
Taiwan	743	18,624	25.07	1047

TLS, total link strength value attributed by VOSviewer.

**Table 9:** The most prolific journals in robotic rehabilitation on stroke survivors’ field throughout 2012–2022.

Journals	No.	C	Average Citation/ Paper	IF	IF without Self-Citations	Publisher
Frontiers in neurology	566	4637	8.19	4.086	3.838	FRONTIERS MEDIA SA
Journal of stroke & cerebrovascular diseases	534	4237	7.93	2.677	2.498	ELSEVIER
Journal of NeuroEngineering and Rehabilitation	484	11,093	22.92	5.208	4.785	BMC
PLOS ONE	466	11,267	24.18	3.752	3.608	PUBLIC LIBRARY SCIENCE
Topics in stroke rehabilitation	451	4938	10.95	2.177	2.113	TAYLOR & FRANCIS LTD
Neurorehabilitation and neural repair	434	11,917	27.46	4.895	4.602	SAGE PUBLICATIONS INC
Disability and rehabilitation	382	4271	11.18	2.439	2.182	TAYLOR & FRANCIS LTD
Neurorehabilitation	303	3556	11.74	1.986	1.919	IOS PRESS
IEEE transactions on neural systems and rehabilitation engineering	296	4926	16.64	4.528	4.167	IEEE-INST ELECTRICAL ELECTRONICS ENGINEERS INC
Archives of physical medicine and rehabilitation	274	6388	23.31	4.060	3.804	W B SAUNDERS CO-ELSEVIER INC

No., number of papers; C, citations; IF, impact factor; BMC, BioMed Central.

**Table 10:** The most influential papers in the field between 2012–2022.

First Author	Title	Journal	IF	C	Ref.
Jovin (2015)	Thrombectomy within 8 Hours after Symptom Onset in Ischemic Stroke	New England Journal of Medicine	176.079	2925	[59]
Vos (2020)	Global burden of 369 diseases and injuries in 204 countries and territories, 1990–2019: a systematic analysis for the Global Burden of Disease Study 2019	Lancet	202.731	1500	[60]
Hochberg (2012)	Reach and grasp by people with tetraplegia using a neurally controlled robotic arm	Nature	69.504	1431	[61]
Lefaucheur (2014)	Evidence-based guidelines on the therapeutic use of repetitive transcranial magnetic stimulation (rTMS)	Clinical neurophysiology	4.861	1099	[62]
Winstein (2016)	Guidelines for Adult Stroke Rehabilitation and Recovery A Guideline for Healthcare Professionals From the American Heart Association/American Stroke Association	Stroke	10.170	1036	[21]
Fernando Nicolas-Alonso (2012)	Brain Computer Interfaces, a Review	Sensors	3.847	991	[63]
Anttila (2018)	Analysis of shared heritability in common disorders of the brain	Science	63.714	824	[64]
Xiong (2013)	Animal models of traumatic brain injury	Nature reviews neuroscience	38.755	800	[65]
Polygerinos (2015)	Soft robotic glove for combined assistance and at-home rehabilitation	Robotics and autonomous systems	3.700	742	[66]
Murray (2020)	Global burden of 87 risk factors in 204 countries and territories, 1990–2019: a systematic analysis for the Global Burden of Disease Study 2019	Lancet	202.731	644	[67]

**Table 11:** Average publication years of the most prolific journals in the three evaluated periods.

1975–2000	Average Publication Year	2001–2011	Average Publication Year	2012–2022	Average Publication Year
Stroke	1995.22	Archives of Physical Medicine and Rehabilitation	2005.92	Frontiers in Neurology	2019.55
Archives of Physical Medicine and Rehabilitation	1997.14	Stroke	2006.45	Journal of Stroke & Cerebrovascular Diseases	2018.18
Neurology	1996.21	Neurorehabilitation and Neural Repair	2008	Journal of Neuroengineering and Rehabilitation	2017.5
American Journal of Physical Medicine & Rehabilitation	1996.59	Clinical Rehabilitation	2006.07	Plos One	2016.27
Acta Neurologica Scandinavica	1994.97	Journal of Rehabilitation Medicine	2007.63	Topics in Stroke Rehabilitation	2017.26
Journal of Neurology Neurosurgery and Psychiatry	1995.97	Disability and Rehabilitation	2007.36	Neurorehabilitation and Neural Repair	2016.83
Brain Research	1995.25	Topics in Stroke Rehabilitation	2009.07	Disability and Rehabilitation	2017.67
Clinical Rehabilitation	1998.93	American Journal of Physical Medicine & Rehabilitation	2005.8	Neurorehabilitation	2016.34
Experimental Neurology	1996.42	Experimental Neurology	2007.24	IEEE Transactions on Neural Systems and Rehabilitation Engineering	2017.8
Scandinavian Journal of Rehabilitation Medicine	1991.12	Journal of Neuroscience	2006.83	Archives of Physical Medicine and Rehabilitation	2016.17

## Data Availability

Not applicable.
